# Annexin-A1-Derived Peptide Ac2-26 Suppresses Allergic Airway Inflammation and Remodelling in Mice

**DOI:** 10.3390/cells11050759

**Published:** 2022-02-22

**Authors:** Tatiana Paula Teixeira Ferreira, Fernanda Verdini Guimarães, Yago Amigo Pinho Jannini Sá, Natalia Barreto da Silva Ribeiro, Ana Carolina Santos de Arantes, Vinicius de Frias Carvalho, Lirlândia Pires Sousa, Mauro Perretti, Marco Aurélio Martins, Patrícia Machado Rodrigues e Silva

**Affiliations:** 1 Laboratory of Inflammation, Oswaldo Cruz Institute, FIOCRUZ, Rio de Janeiro 21040-360, Brazil; tatpaula@ioc.fiocruz.br (T.P.T.F.); nandaverdini22@yahoo.com.br (F.V.G.); yagoapjs@gmail.com (Y.A.P.J.S.); natalia_bsribeiro@hotmail.com (N.B.d.S.R.); ac_sda@yahoo.com.br (A.C.S.d.A.); vfrias@ioc.fiocruz.br (V.d.F.C.); mmartins@ioc.fiocruz.br (M.A.M.); 2 Faculty of Pharmacy, Federal University of Minas Gerais, Belo Horizonte 31270-901, Brazil; lipsousa72@gmail.com; 3 Department of Biochemical Pharmacology, The William Harvey Research Institute, Queen Mary University of London, London EC1M 6BQ, UK; m.perretti@qmul.ac.uk

**Keywords:** lung, inflammation, remodelling, allergic asthma, therapy, peptide Ac2-26

## Abstract

Annexin-A1 (AnxA1) and its N-terminal derived peptide Ac2-26 regulate the inflammatory response in several experimental models of disorders. This study evaluated the effect of endogenous AnxA1 and its N-terminal peptide Acetyl 2-26 (Ac2-26) on allergic asthma triggered by house dust mite (HDM) extract in mice. *ANXA*1^−/−^ and wildtype (WT) mice were exposed to intranasal instillation of HDM every other day for 3 weeks, with analyses performed 24 h following the last exposure. Intranasal administration of peptide Ac2-26 was performed 1 h before HDM, beginning 1 week after the initial antigen application. *ANXA*1^−/−^ mice stimulated with HDM showed marked exacerbations of airway hyperreactivity (AHR), eosinophil accumulation, subepithelial fibrosis, and mucus hypersecretion, all parameters correlating with overexpression of cytokines (IL-4, IL-13, TNF-α, and TGF-β) and chemokines (CCL11/eotaxin-1 and CCL2/MCP-1). Intranasal treatment with peptide Ac2-26 decreased eosinophil infiltration, peribronchiolar fibrosis, and mucus exacerbation caused by the allergen challenge. Ac2-26 also inhibited AHR and mediator production. Collectively, our findings show that the AnxA1-derived peptide Ac2-26 protects against several pathological changes associated with HDM allergic reaction, suggesting that this peptide or related AnxA1-mimetic Ac2-26 may represent promising therapeutic candidates for the treatment of allergic asthma.

## 1. Introduction

Allergic asthma is one of the most common respiratory disorders and affects approximately 330 million people worldwide [1]. Studies have reported asthma prevalence rates, ranging between 1% and 18% in the general population of various countries [2]. Recurrent and persistent asthma is driven by a T-helper 2 (Th2)-polarised immune response, eosinophilic inflammation, and airway remodelling, and it is characterised by subepithelial fibrosis and increased levels of collagen and other extracellular matrix (ECM) proteins [3]. Moreover, excessive mucus secretion and hyperplasia/hypertrophy of smooth muscle markedly contribute to the thickening of the airway walls and airflow limitations [4].

Glucocorticoids have broad-ranging and potent anti-inflammatory and immunomodulatory effects [5]. Annexin (Anx)A1 is a 37 kDa glucocorticoid-induced protein that reproduces many of the pharmacological actions of glucocorticoids by binding to phospholipids in a Ca^2+^-dependent manner, inhibiting cytosolic phospholipase A_2_ activity and preventing the synthesis of arachidonic acid-derived metabolites. This protein is widely distributed throughout the body and is constitutively produced by monocytes, macrophages, neutrophils, T cells [6,7,8], and mast cells [9]. Significant modulatory effects of AnxA1 occur through binding to formyl peptide receptors (FPRs), which are G-protein-coupled receptors (GPCRs), and studies have revealed that AnxA1 has a greater binding affinity to FPR2/ALX receptors [10].

Notably, the data gathered from the literature attest that the N-terminal portion of the AnxA1 peptide (i.e., the Ac2-26 peptide) is capable of reproducing the anti-inflammatory actions of the full-length protein [11], showing great promise in several experimental models of disease [10,12,13,14]. In the context of lung inflammation, this peptide attenuates tissue recruitment of eosinophil, protein extravasation, and mediator release in both rats [15] and mice [16]. However, the effect of such treatment on tissue remodelling and mucus hypersecretion has not yet been explored.

Experimental models of asthma were previously based on sensitisation of rodents to an innocuous protein antigen ovalbumin (OVA), which was conjugated with adjuvant aluminium hydroxide, followed by repeated exposures of OVA into the airways [17]. Alternatively, OVA has been replaced by house dust mite (HDM), a naturally occurring indoor aeroallergen source transmitted by the airways that results in a Th2 adaptative immune response with elevated levels of IgE, but whose complexity may influence the mechanisms that underlie the processes of sensitisation, inflammation, and remodelling [18].

The current study investigated the effect of the peptide Ac2-26 in experimental models of allergic asthma triggered by HDM or OVA in mice, by analysing lung function, leukocyte recruitment, and the extent of tissue remodelling and mucus production.

## 2. Methods

### 2.1. Animals

Female and male wildtype (WT) Balb/c mice (18–20 g) and AnxA1-knockout mice (*ANXA1*^−/−^) on a Balb/c background were used [19]. No sex-dependent differences were observed in the experiments. In peptide Ac2-26 treatment experiments, male Balb/c mice were used. All animals were obtained from the Oswaldo Cruz Foundation breeding facility, being kept in a 12 h light/dark cycle and fed water and food ad libitum. All the procedures involving the care and use of laboratory animals were examined and approved by the Animal Ethics Committee of the Oswaldo Cruz Institute (L-001/19).

### 2.2. Sensitization, Allergen Challenge, and Treatment

**Figure 1:**
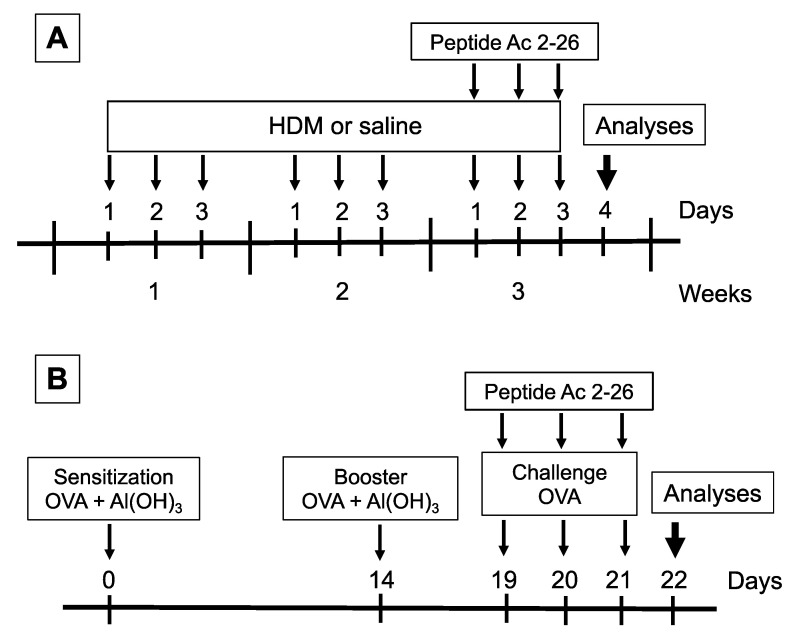
Schematic protocol flowchart of models of allergic asthma and treatment with peptide Ac2-26. (**A**) HDM model mice received a series of intranasal provocations with HDM (15 μg/25 μL) 3 days a week for a period of 3 weeks. The peptide Ac2-26 was administered intranasally (200 μg/mouse), 1 h before the challenges given on the third week. Analyses were performed 24 h after the last challenge. (**B**) OVA model mice were sensitised at days 0 and 14 and subjected to three consecutive daily intranasal injections of OVA (25 μg/25 μL), at days 19, 20, and 21 post sensitisation. Peptide Ac2-26 was given intranasally (200 μg/mouse), 1 h before each OVA stimulation. Analyses were performed 24 h after the last challenge.

Two allergens were used to induce allergic asthma in mice. For the HDM model, under the condition of light anaesthesia (isoflurane) (Cristália, São Paulo, Brazil), mice received an intranasal (i.n.) instillation of 15 μg (total protein) of *Dermatophagoides pteronyssinus* (HDM) extract (GREER, North Carolina, USA) in 25 μL of sterile 0.9% NaCl (saline), up to a frequency of 3 days per week for a period of 3 weeks (Figure 1A) [20]. HDM endotoxin content was 0.59 × 10^3^ EU/mg. For the ovalbumin (OVA) model, mice received a subcutaneous injection of a mixture containing 50 µg of OVA (Grade V; Sigma-Aldrich, Milwaukee, WI, USA) and 5 mg Al(OH)_3_, in saline, before being boosted 14 days later. From day 19 to 21 post sensitization, under isoflurane anaesthesia (Cristália, São Paulo, Brazil), mice were challenged with OVA (25 µg/25 µL, i.n.) (Figure 1B) [21]. Control mice were challenged with 25 µL of saline following each protocol described above. As shown in Figure 1, 1 h before HDM (Figure 1A) or OVA (Figure 1B) challenge, animals under isoflurane volatile anaesthesia (Cristália, São Paulo, Brazil) were treated i.n. with the peptide Ac2-26 (Ac-AMVSEFLKQAWFIENEEQEYVQTVK) (200 µg/mouse) (Cambridge Research Biochemicals, Cleveland, UK) [22]. Analyses were performed 24 h after the last challenge for both models employed.

### 2.3. Respiratory Mechanics

Mice were monitored using an invasive whole-body plethysmograph, when airflow and transpulmonary pressure were registered with a Pulmonary Mechanics Computer (DSI, Minneapolis, MN, USA) using, as parameters, airway resistance (cm H_2_O·s/mL) and lung elastance (cm H_2_O/mL). Mice were anesthetised intraperitoneally (i.p.) with pentobarbital (Sigma Aldrich, São Paulo, Brazil) (60 mg/kg), tracheostomised, and mechanically ventilated. Changes in resistance and elastance were taken at baseline, and airway hyperreactivity (AHR) was determined as changes in airway function after aerosolised methacholine (3–81 mg/mL) (Sigma Aldrich, São Paulo, Brazil) as previously reported [23].

### 2.4. Bronchoalveolar Lavage Fluid

Mice were killed by an overdose of sodium pentobarbital (500 mg/kg, i.p.; Sigma Aldrich, São Paulo, Brazil). The airways were washed twice with 750 µL of EDTA 10 mM in PBS 1×, and the fluid was centrifuged at 400× *g* for 10 min at 4 °C. The cell pellet was resuspended in 250 µL of EDTA 10 mM in PBS 1× and enumerated in a Neubauer chamber after dilution with Türk solution. The differential cell counts were carried out on May–Grunwald–Giemsa-stained cytospin preparations under an oil immersion objective using a light microscope (BX50; Olympus, Center Valley, PA, USA).

### 2.5. Histological Analyses

The left lung was fixed in Millonig’s buffer solution (pH = 7.4) with 4% paraformaldehyde for 48 h to preserve tissue architecture. Briefly, samples were embedded in paraplast (Sigma-Aldrich, São Paulo, Brazil), and 4 µm thick sections were stained with Sirius Red (pH = 10.2) for eosinophil infiltrates in the peribronchiolar area, with determinations made in six randomly selected fields at a magnification of ×1000 and expressed as eosinophils per unit area (μm^2^). Histologic sections were stained with periodic acid–Schiff (PAS) for measuring mucus production and Gömöri trichrome (GT) to quantify the total deposition of ECM. The area of peribronchiolar staining (i.e., between the alveolar septum and airway epithelium of 10 distal airways by lung section) was outlined and quantified for the total deposition of ECM. The evaluation was made in an image analyser system (Image-Pro^®®^ Plus, 4.1; Media Cybernetics, Houston, TX, USA), using digitalised images obtained from a light microscope at a magnification of ×400. All analyses were performed under blinded conditions.

### 2.6. ELISA Analysis

CCL2/MCP-1, CCL11/Eotaxin-1, TGF-β, IL-4, IL-13, and TNF-α levels were quantified in the right-lung tissue samples, which were homogenised in 1 mL of PBS 1× containing 0.05% Triton X-100 and protease inhibitor (cOmplete–Roche, Mannheim, Germany). Samples were quantified using commercially available kits (DuoSet system) following the manufacturer’s instructions (R&D Systems, Minneapolis, MN, USA).

### 2.7. Statistical Analysis

Data analyses were performed with a statistical software package (Prism version 7.0, Graph-Pad Software, San Diego, CA, USA). Data were expressed as the mean ± SEM. The tests were carried out using one way or two-way ANOVA followed by Newman–Keuls test, with *p* < 0.05 considered statistically significant.

## 3. Results

### 3.1. Lack of AnxA1 Expression Exacerbates Lung Function in HDM-Exposed Mice

**Figure 2:**
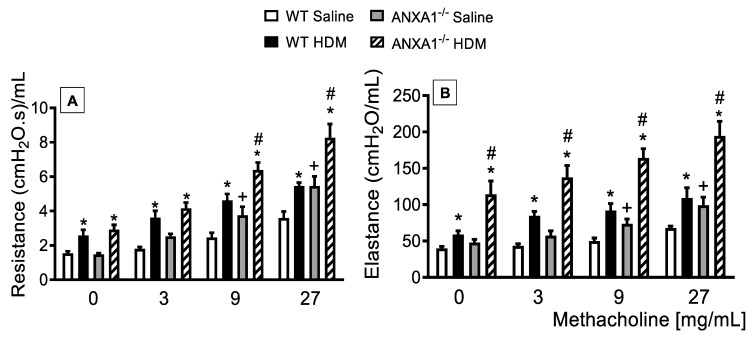
Analysis of the effect of endogenous AnxA1 knockout on HDM-induced airway hyperreactivity in mice, measured as resistance (**A**) and elastance (**B**) induced by provocation with increasing concentrations of methacholine. Animals instilled with saline were used as controls. Analyses were performed 24 h after the last HDM or saline challenge. Values represent the mean ± SEM from 6–7 animals per group. * *p* < 0.05 as compared to their respective saline control groups; + *p* < 0.05 as compared to the saline-challenged WT group; # *p* < 0.05 as compared to the HDM-challenged WT group.

To assess putative changes in the airway function following HDM challenge, under conditions of abolished *ANXA1* gene expression, we undertook invasive measurements of airway resistance and lung elastance responses to inhaled methacholine (3–27 mg/mL), which was evaluated 24 h after the last allergen provocation. WT and *ANXA1*^−/−^ mice exposed to HDM showed an increase in the baseline levels of airways resistance and lung elastance when compared to WT controls (Figure 2A,B, respectively). Antigen challenge with HDM led to AHR, as attested by increased airway resistance and lung elastance responses after methacholine, relative to untreated control mice. On the other hand, HDM-challenged *ANXA1*^−/−^ mice exhibited higher levels of airways resistance and lung elastance relative to WT animals (Figure 2A,B, respectively).

### 3.2. Lack of AnxA1 Expression Exacerbates Airways Inflammation, Histological Changes, and Mediator Production in HDM-Exposed Mice

**Figure 3:**
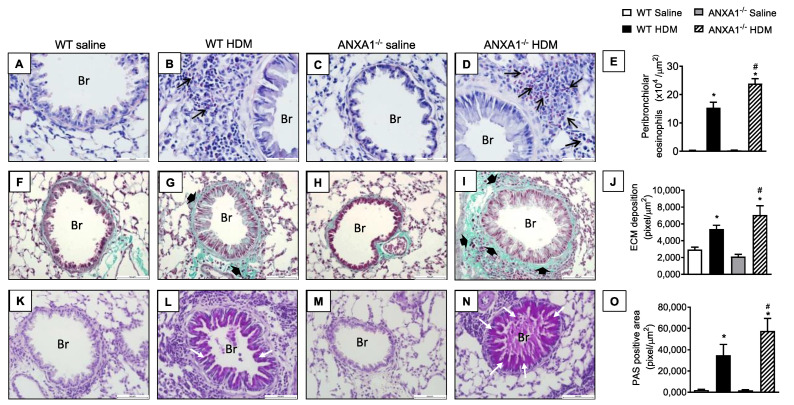
Analysis of the effect of endogenous AnxA1 knockout on HDM-induced lung inflammation in mice. Photomicrographs are representative of lung tissue sections showing peribronchiolar eosinophil accumulation (**A**–**D**) (Sirus Red stain), subepithelial fibrosis (**F**–**I**) (Gömöri trichrome stain), and goblet-cell hyperplasia/mucus production (**K**–**N**) (periodic acid–Schiff stain). Quantitative assessments of eosinophil infiltration (**E**), extracellular matrix deposition (**J**), and mucus production (**O**) were performed in lung tissue, as described in Section 2. Animals instilled with saline were used as controls. Analyses were performed 24 h after the last HDM or saline challenge. Scale bar = 100 µm. Black arrows = eosinophils; black arrowheads = extracellular matrix; white arrows = mucus. Br = bronchioles. Values represent the mean ± SEM from 6–7 animals per group. * *p* < 0.05 as compared to their respective saline-challenged groups; ^#^ *p* < 0.05 as compared with HDM-challenged WT group.

When exposed to HDM, WT mice showed marked inflammatory cell infiltration around the bronchioles composed mostly by eosinophils compared to negative controls (Figure 3A,B, respectively). This response was also noted in *ANXA1*^−/−^ HDM-challenged mice, with greater intensity compared to WT HDM-challenged mice (Figure 3B,D, respectively). No significant differences in eosinophil infiltration were found between *ANXA1*^−/−^ and WT saline-exposed mice (Figure 3A,C, respectively). Quantitative data on eosinophil enumeration are shown in Figure 3E.

The staining of lung sections with Gömöri trichrome, a classical stain for ECM components, revealed an increase in ECM deposition in the peribronchiolar areas in WT mice exposed to HDM compared to the negative controls (Figure 3F,G respectively). *ANXA1*^−/−^ mice challenged with HDM showed a similar response, but greater Gömöri trichrome staining was noted (Figure 3I). *ANXA1*^−/−^ mice exposed to saline did not show alteration in Gömöri trichrome stain compared to WT controls (Figure 3H). Morphometric analyses are shown in Figure 3J.

Representative photomicrographs of lung sections stained with PAS showed that there is no mucus production detected in saline-instilled WT or knockout mice (*ANXA1*^−/−^) (Figure 3K,M, respectively), under conditions where HDM-challenged WT mice exhibited hyperplasia of goblet cells and mucus secretion in the airways (Figure 3L). HDM-stimulated *ANXA1*^−/−^ mice showed a higher degree of goblet cell hyperplasia and mucus hypersecretion compared to WT mice (Figure 3N). Morphometric analyses are presented in Figure 3O.

**Figure 4:**
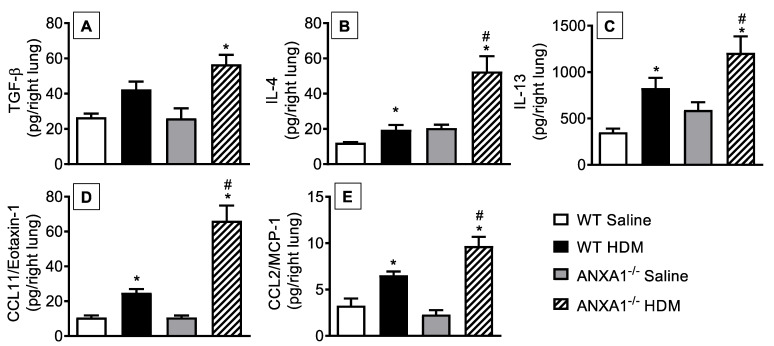
Analysis of the effect of endogenous AnxA1 knockout on HDM-induced cytokine and chemokine production in the lungs of mice. TGF-β (**A**), IL-4 (**B**), IL-13 (**C**), CCL11/eotaxin-1 (**D**), and CCL2/MCP-1 (**E**) were measured in lung tissue homogenates from saline- and HDM-challenged WT and *ANXA1*^−/−^ mice. Animals instilled with saline were used as controls. Analyses were performed 24 h after the last HDM or saline-challenged. Values represent the mean ± SEM from 6–7 animals per group. * *p* < 0.05 as compared to their respective saline-challenged groups; ^#^ *p* < 0.05 as compared to HDM-challenged WT group.

Levels of cytokines such as TGF-β (Figure 4A), IL-4 (Figure 4B), and IL-13 (Figure 4C) and of chemokines CCL11/eotaxin-1 (Figure 4D) and CCL2/MCP-1 (Figure 4E) were increased in the lung tissue of HDM-exposed WT mice compared to the saline-stimulated WT group. *ANXA1*^−/−^ mice challenged with the antigen showed higher levels of these proinflammatory mediators relative to HDM-challenged WT controls (Figure 4).

### 3.3. Effect of Peptide Ac2-26 on Lung Remodelling and Mucus Hypersecretion in OVA-Challenged Mice

**Figure 5:**
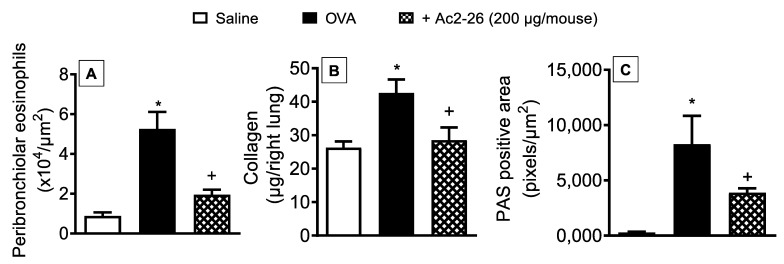
Intranasal peptide Ac2-26 reduces airway remodelling caused by OVA in sensitised mice. Peribronchiolar eosinophil infiltration (**A**), collagen deposition (**B**), and goblet-cell hyperplasia/mucus production (**C**) in the lungs of OVA-challenged mice and treated with peptide Ac2-26 (200 µg/mouse, i.n.). Animals instilled with saline were used as controls. Quantitative assessment of eosinophil infiltration (Sirius Red stain), collagen deposition (Sircol technique), and mucus production (periodic acid–Schiff stain) were performed in the lung tissue as described in Section 2. Analyses were performed 24 h after the last OVA challenge. Values represent the mean ± SEM from six animals per group. * *p* < 0.05 as compared to the saline-challenged group; + *p* < 0.05 as compared to the OVA-challenged group.

To determine whether the peptide Ac2-26 could exert a protective effect on OVA-induced lung morphological changes, the compound was administered, intranasally, 1 h before OVA provocations, and lung histological sections were harvested 24 h after the last challenge. As illustrated in Figure 5, OVA-challenged mice exhibited peribronchiolar eosinophilic inflammatory infiltration (Figure 5A), a twofold increase in the amount of lung collagen (Figure 5B), and mucus hypersecretion (Figure 5C), as compared to saline-challenged animals. Treatment with peptide Ac2-26 reduced the eosinophil recruitment, collagen deposition, and mucus secretion in the lungs of OVA-sensitized mice (Figure 5).

### 3.4. Effect of Peptide Ac2-26 on Lung Changes in HDM-Exposed Mice

**Figure 6:**
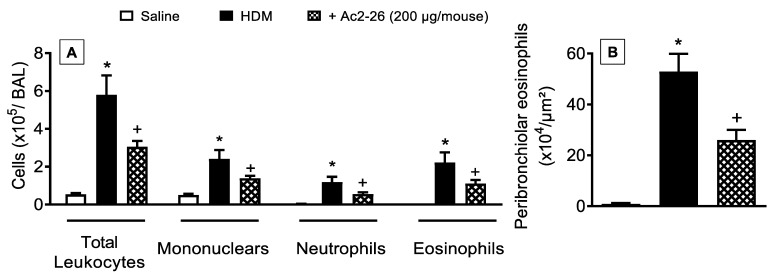
Intranasal peptide Ac2-26 reduces leukocyte recruitment into the airways after HDM challenge. (**A**) Leukocyte accumulation in the bronchoalveolar lavage (BAL) and (**B**) peribronchiolar eosinophil infiltration in the lung tissue of HDM-challenged mice and treated with peptide Ac2-26 (200 µg/mouse, i.n.). Animals instilled with saline were used as controls. Quantitative assessments of eosinophil levels were performed in lung tissue as described in Section 2. Analyses were performed 24 h after the last HDM or saline challenge. Values represent the mean ± SEM from eight animals per group. * *p* < 0.05 as compared to the saline-challenged group; + *p* < 0.05 as compared to the HDM-challenged group.

Next, we examined the interventional effect of the peptide Ac2-26 on HDM-triggered allergic inflammation in the lung. As compared to sham-challenged control animals, mice stimulated by HDM showed markedly greater total leukocyte numbers, which was accounted for by an increase in the number of mononuclear cells, neutrophils, and eosinophils. All these changes were sensitive to treatment with peptide Ac2-26 (Figure 6).

**Figure 7:**
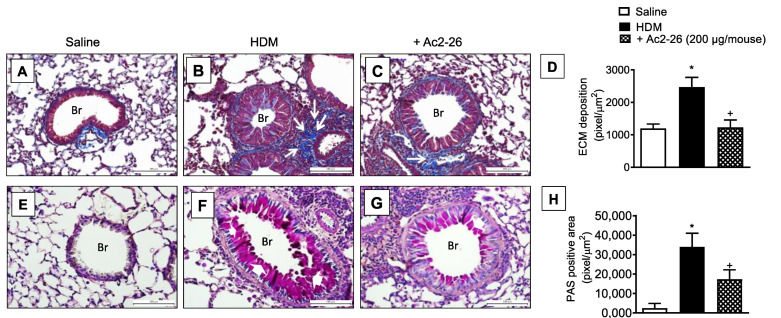
Intranasal peptide Ac2-26 reduces the airway remodelling and mucus production in the lungs following HDM stimulation. Photomicrographs of extracellular matrix deposition (**A**–**C**) and mucus production (**E**–**G**) in the lungs of saline- or HDM-challenged mice and treated with peptide Ac2-26 (200 µg/mouse, i.n.). Quantitative assessments of subepithelial fibrosis (**D**) and mucus production (**H**) were performed after staining with Gömöri trichrome and periodic acid–Schiff, respectively. Animals instilled with saline were used as controls. The analyses were performed 24 h after the last HDM or saline challenge. Scale bar = 100 µm. White arrows = extracellular matrix; white arrowheads = mucus; Br = bronchioles. Values represent the mean ± SEM from eight animals per group. * *p* < 0.05 as compared to the saline-challenged group; + *p* < 0.05 as compared to the HDM-challenged group.

**Figure 8:**
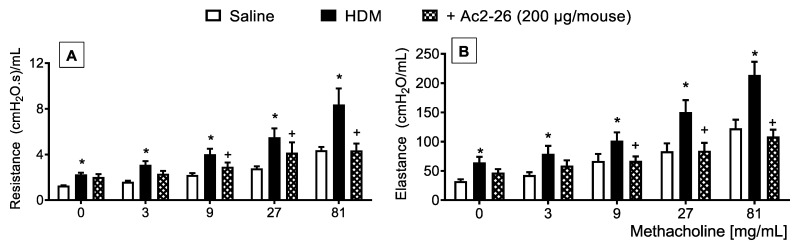
Effect of peptide Ac2-26 on methacholine-induced changes in airway resistance and lung elastance in HDM-challenged mice. Airway hyperreactivity was measured as resistance (**A**) and elastance (**B**) induced by provocation with increasing concentrations of methacholine in HDM-challenged mice and treated with peptide Ac2-26 (200 µg/mouse, i.n.). Animals instilled with saline were used as controls. The analyses were performed 24 h after the last HDM or saline challenge. Values represent the mean ± SEM from eight animals per group. * *p* < 0.05 as compared to the saline-challenged group; + *p* < 0.05 as compared to the HDM-challenged group.

In HDM-challenged mice, the mucus overproduction (Figure 7F) and peribronchiolar fibrosis (Figure 7B) were found in HDM-challenged relative to sham-challenged mice (Figure 7A,E, respectively), these phenomena were markedly inhibited by peptide Ac2-26 (Figure 7C,G, respectively). Morphometric analyses are shown in Figure 7D,H. Ac2-26 inhibited AHR in HDM-challenged mice, concerning both airway resistance and lung elastance (Figure 8).

**Figure 9:**
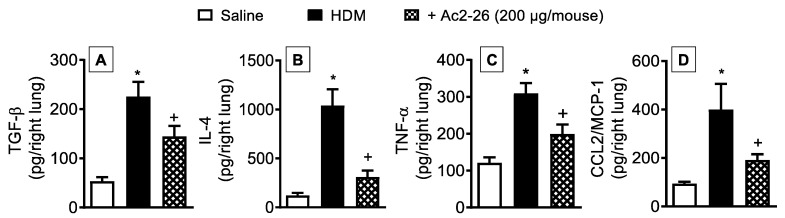
Intranasal peptide Ac2-26 reduces proinflammatory cytokine and chemokine levels in the lung samples following HDM stimulation. Production of cytokines and of chemokine in the lungs of HDM-challenged mice and treated with peptide Ac2-26 (200 µg/mouse, i.n.). TGF-β (**A**), IL-4 (**B**), TNF-α (**C**), and CCL2/MCP-1 (**D**) were measured in lung tissue homogenates from saline- and HDM-challenged mice. Animals instilled with saline were used as controls. Analyses were performed 24 h after the last HDM or saline challenge. Values represent the mean ± SEM from eight animals per group. * *p* < 0.05 as compared to the saline-challenged group; + *p* < 0.05 as compared to the HDM-challenged group.

Quantification of proinflammatory cytokines and chemokines in the lungs of HDM-stimulated mice revealed an increase in the levels of TGF-β (Figure 9A), IL-4 (Figure 9B), TNF-α (Figure 9C), and CCL2/MCP-1 (Figure 9D), responses which were partially reduced by treatment with peptide Ac2-26 as compared to HDM-challenged untreated animals. 

## 4. Discussion

In the present study, we evaluated the potential ability of AnxA1 to modulate lung inflammation and remodelling in two distinct models of allergic asthma in mice. We demonstrated that, when stimulated with HDM extract, AnxA1 knockout mice showed exacerbation of AHR and presented increased pathological in the lung, monitored as eosinophilic inflammatory infiltrate, mucus hypersecretion, subepithelial fibrosis, and collagen deposition; such changes were associated with overproduction of proinflammatory cytokines/chemokines, as compared to challenged WT animals. Moreover, local treatment with the AnxA1 mimetic peptide Ac2-26 protected against AHR, subepithelial fibrosis, and mucus hypersecretion triggered by either HDM or OVA. Thus, our findings corroborate the proposition that the AnxA1 peptide Ac2-26 holds promise as an innovative therapy for the treatment of asthma.

AnxA1 is a protein that is transcribed following binding of the glucocorticoid–glucocorticoid receptor (GR) complex to the glucocorticoid response element (GRE) in the promoter regions of glucocorticoid-regulated genes [24]. As such, this protein is engaged in natural and glucocorticoid-driven processes of inhibition and resolution of inflammation [5,25]. Interestingly, AnxA1 levels are higher in the plasma of asthmatic patients, as well as in the BAL and plasma of asthmatic mice [26], which is likely to represent an attempt of the host to modulate the over-reactive inflammatory response. On the other hand, plasma levels of AnxA1 were lower in patients with asthma exacerbation than in patients with stable asthma [26], thus indicating a correlation between lower levels of the protein AnxA1 and bronchial asthma severity. In view of this controversy, prior reports showed that, in the BAL of asthmatics [27] and in smokers [28], AnxA1 (37kDa) can be proteolyzed to a number of fragments (i.e., 33 kDa form) that lose their ability to reduce inflammation when compared to the native molecule or its N-terminal derived peptides [29]. This suggests that, although AnxA1 levels may be high under some lung pathological conditions, it will be cleaved and rendered inactive, thus leading to chronic and uncontrolled inflammation.

To better understand this point, we evaluated the effect of AnxA1 depletion by assessing the impact of allergen-induced pathological changes in the lung of actively sensitised AnxA1-null mice. As compared to HDM-challenged WT mice, AnxA1-null mice presented exacerbation of HDM-induced eosinophilic inflammation in the lungs, in parallel with higher levels of proinflammatory cytokines IL-4, IL-13, and TGF-β and the chemokines CCL11/eotaxin-1 and CCL2/MCP-1; all these outcomes were matched by an aggravation of AHR. This corroborates the notion that lack of AnxA1 leads to a more pronounced Th2 phenotype of activated Th cells relative to WT animals [30]. Moreover, these findings are in line with previous research showing exacerbation of AHR and lung leukocyte infiltration in AnxA1-deficient sensitised mice subjected to OVA provocation [16].

Our study demonstrates for the first time that the AnxA1-null mice showed aggravation of HDM-challenge-evoked airway remodelling as attested by increased peribronchiolar fibrosis and excess ECM deposition. Evidence suggests that the endogenous protein AnxA1 may downregulate pulmonary fibrosis, since AnxA1-null mice show excess ECM deposition after stimulation with bleomycin and crystalline silica particles [22,31]. Furthermore, the RNAi-mediated knockdown of *ANXA1* expression in normal lung fibroblasts increased TNF-α-stimulated proliferation and IL-6 production in vitro [32].

Our findings revealed that AnxA1-knockout mice stimulated with HDM showed higher levels of Th2-type cytokines (IL-4, IL-13, and TGF-β) and chemokines (CCL11/eotaxin-1 and CCL2/MCP-1) as compared to HDM-challenged WT mice. The elevated levels of proinflammatory mediators correlated with exacerbation of eosinophil accumulation and subsequent airway remodelling, suggesting a beneficial role of AnxA1 or its mimetic peptide in the treatment of severe asthma. In many in vitro and in vivo systems, the anti-inflammatory effects of AnxA1 have been reproduced by compounds derived from the N-terminal region of the protein, including the peptide Ac2-26 [5,33]. Herein, we demonstrated that intranasal treatment with peptide Ac2-26 inhibited OVA-induced tissue eosinophil infiltration, tissue remodelling, and mucus production. Moreover, peptide Ac2-26 inhibited HDM-evoked accumulation of eosinophils, neutrophils, and mononuclear cells in the airway lumen, as well as abrogated subepithelial infiltration of eosinophils, thereby reducing the accompanying mucus overproduction and AHR. In accordance with the HDM findings, the peptide also led to a significant decrease in tissue inflammation caused by OVA challenge, decreasing eosinophil accumulation and mucus hypersecretion. These data are supported by the observation that the cell-permeable protein Tat-AnxA1 reduces the inflammatory response in BAL and lung, as well as the AHR after allergen challenge [34]. Peptide Ac2-26 also decreased the production of Th2-type cytokine IL-4 and of the chemokine CCL2/MCP-1 in the lung tissue after HDM. Inhibition of tissue eosinophil recruitment in the lungs by Ac2-26 has been associated with downstream reduced secretion of pivotal eosinophil chemoattractant and activators, including eotaxin-1 and PGD_2_, but not with blockade of eosinophil chemotaxis [15,35]. Importantly, local administration of Ac2-26 reduced mucus overproduction and peribronchiolar fibrosis stimulated by HDM or OVA as attested by quantitative histological evaluations. AnxA1 and its derived peptide Ac2-26 were reported to exert their effects through activation of the formyl peptide receptor type 1 (FPR1) and type 2 (FPR2) [10,36], present on the cellular membrane of leukocytes [37]. Previous work showed that AnxA1 engagement to FPR2 activated the p38 MAPK/MAPKAPK/Hsp27 signalling cascade, which led to the production of the anti-inflammatory cytokine IL-10 [38]. Peptide Ac2-26 was also reported to activate the JNK/caspase-3 pathway leading to leukocyte apoptosis [39]. Moreover, activation of FPR2 and the major regulator of cellular metabolism AMPK led to changes of immune cells that can downregulate their inflammatory activity [40].

Previous reports have described the inhibitory properties of peptide Ac2-26 in models of chronic lung disease, a phenomenon accounted for by the suppression of profibrotic cytokine production including TNF-α and TGF-β [22,31]. Aligned with these observations, our results indicate that the production of TNF-α and TGF-β in the lungs of mice stimulated by HDM is abrogated by the peptide. Furthermore, overexpression of AnxA1 attenuated TGF-β-evoked increases in the mRNA levels of α-smooth muscle actin (α-SMA) and collagen type 1A1 [41]. Altogether, these findings indicate that Ac2-26 may have reduced fibroblast activation and differentiation in HDM-stimulated allergic asthma, with consequent inhibition of ECM deposition. Giving support to this interpretation, we demonstrated that peptide Ac2-26 suppressed IL-13- and TGF-β-induced collagen production and MCP-1 secretion in mouse lung fibroblasts in vitro [22]. Strikingly, lung fibroblasts express mRNA for FPR1 and FPR2 under basal conditions, and there is an increase in mRNA expression for both receptors after stimulation with IL-13 [22]. Using lung fibroblasts from mice lacking the gene for *FPR1* and *FPR2*, we noted that FPR1 plays an important role in the suppressive effect of peptide Ac2-26 on collagen and CCL2/MCP-1 generation by lung fibroblasts, while FPR2 seems to be important only for collagen production [22].

Metalloproteinases are zinc-dependent endopeptidases that degrade all components of the ECM and possess antifibrotic activities in murine pulmonary fibrosis, especially matrix metalloproteinase-1 (MMP-1), which has the potential to limit fibrotic responses to injury [42]. Relevantly, peptide Ac2-26 was found to stimulate MMP-1 secretion by synovial fibroblasts in vitro [43]. The mechanism underlying the suppressive effect of the peptide Ac2-26 on allergen-induced inflammation and remodelling is incompletely understood; therefore, further investigations are needed in order to clarify this point.

In conclusion, we demonstrate that local treatment with the AnxA1-derived peptide Ac2-26 prevents allergen-induced eosinophilic infiltration, AHR, and peribronchiolar fibrosis with a mechanism of action closely related to down-regulation of Th2 cytokines (e.g., IL-4 and IL-13) and chemokines (e.g., CCL-11/eotaxin-1 and CCL2/MCP-1). These results may form the basis for further work on the development of AnxA1-mimetic peptides as innovative therapy for allergic asthma.

## Data Availability

All data analysed during this study are included in this published article. Specific requests can be made via e-mail to the corresponding authors.

## Abbreviations

α-SMA	α-smooth muscle actin
AHR-	airway hyperreactivity
AnxA1	annexin-A1
CCL2/MCP-1	CC ligand-2/ monocyte chemoattractant protein-1
CCL11/eotaxin1	CC ligand-11/eotaxin-1
HDM	house dust mite
ECM	extracellular matrix
EDTA	ethylenediaminetetraacetic acid
GR	glucocorticoid receptor
GRE	glucocorticoid response element
GT	Gömöri trichrome
MMP-1	matrix metalloproteinase-1
PAS	periodic acid–Schiff
PBS	phosphate-buffered saline
TGF-β	transforming growth factor-β1
TNF-α	tumour necrosis factor-α
WT	wildtype
